# Dexmedetomidine in Healthy Dogs: Impact on Electrocardiographic Parameters

**DOI:** 10.1155/vmi/9947159

**Published:** 2025-08-08

**Authors:** Guilherme Andraus Bispo, Thais Cabral de Oliveira, Marcela Fernanda Moretti, Matheus Fujimura Soares, Lais Calazans Menescal Linhares, Natália Sena Nascimento de Souza, Ana Kelly Sousa da Costa, Marilda Onghero Taffarel, Wagner Luís Ferreira, Paulo Sergio Patto dos Santo

**Affiliations:** ^1^School of Veterinary Medicine (FMV), São Paulo State University (UNESP), Araçatuba, São Paulo, Brazil; ^2^School of Agricultural and Veterinary Sciences (FCAV), São Paulo State University (UNESP), Jaboticabal, Brazil; ^3^State University of Maringá (UEM), Umuarama, Paraná, Brazil

**Keywords:** alpha-2-agonist, atrioventricular block, bradycardia, heart rate variability

## Abstract

**Objective:** To evaluate whether intravenous administration of dexmedetomidine alters the electrocardiographic parameters of healthy dogs.

**Animals:** 22 healthy dogs.

**Study Design:** prospective blind clinical trial.

**Materials and Methods:** The dogs underwent electrocardiographic monitoring at two moments: baseline moment (MB), subjected to tranquilization with butorphanol (0.25 mg/kg/IM), and dexmedetomidine moment (MDEX), after 10 min of intravenous administration of dexmedetomidine (bolus of 2 μg/kg, in 2 minutes, followed by continuous infusion at a rate of 2 μg/kg/hour).

**Results:** After intravenous administration of dexmedetomidine, sinus bradycardia predominated. Regarding electrocardiographic parameters, there was a significant increase in the duration of the P wave (44; 51 ms), QRS complex (62; 67 ms), PR interval (95; 126 ms), and QT interval (207; 252 ms) and reduction in the QTc interval (249; 226 ms), without significant change in systolic, mean, and diastolic blood pressure.

**Conclusion:** Intravenous administration of dexmedetomidine in healthy dogs results in increased parasympathetic tone with significant reduction in heart rate and increased conduction time of electrical impulses in the myocardium.

## 1. Introduction

Dexmedetomidine is a drug belonging to the class of α-2 receptor agonists and has been widely used in short-term outpatient procedures as it provides sedation and analgesia [1, 2]. In the context of balanced anesthesia, it is used as a preanesthetic medication or adjuvant during anesthetic maintenance, being responsible for providing a significant reduction in the requirement for general anesthetics [3–6].

The literature presents a wide range of doses for intramuscular administration of dexmedetomidine [7, 8] or intravenously in the form of bolus or continuous rate infusion (CRI), with rates between 0.5 and 5.0 μg/kg/hour [6, 9, 10].

The analgesic effect associated with dexmedetomidine comes from the stimulation of α-2 receptors in the central nervous system, in a region called *locus coeruleus* [11, 12]. van Oostrom et al. [13] demonstrated that the analgesic effect of IC dexmedetomidine was only achieved with the administration of higher rates (3 μg/kg/hour) in relation to the rate required to obtain sedation in dogs (1 μg/kg/hour). Corroborating these findings, Alves et al. [10], in a pharmacokinetic study, found that the administration of intravenous dexmedetomidine (bolus 2 μg/kg in 2 minutes) provides maximum sedative effect after 12 min.

However, some undesirable effects on the cardiovascular system are observed after the administration of dexmedetomidine, which limits its full acceptance in the anesthetic routine of dogs [14]. One of the effects of this drug is an increase in blood pressure, due to the interaction of the agonist with α-2 receptors in vascular smooth muscle, promoting an increase in peripheral vascular resistance (PVR). [15, 16]. As a result, reflex bradycardia occurs [17, 18] and reduced cardiac output [19]; Fernández-Parra et al. 2021. In addition, dexmedetomidine promotes direct central vagal stimulation, contributing to increased parasympathetic tone on the heart [20].

It is known that, in pediatric human patients, dexmedetomidine promotes depression of sinus and atrioventricular node functions, without affecting the wave intervals of the electrocardiogram. [21]. In felines, dexmedetomidine had no direct effect on sinus and atrioventricular node refractoriness [17]. However, the presence of atrioventricular conduction blocks and sinus arrests was observed in dogs administered dexmedetomidine (0.5 μg/kg/IM) associated with methadone or midazolam [22].

The aim of this study was to evaluate whether intravenous administration of dexmedetomidine (bolus of 2 μg/kg, followed by continuous infusion at a rate of 2 μg/kg/hour) affects electrocardiographic changes in healthy dogs. The hypothesis is that at the proposed dose, dexmedetomidine results in bradycardia and consequently changes in the electrocardiographic parameters evaluated.

## 2. Materials and Methods

### 2.1. Animals

The study was approved by the Ethics Committee on the Use of Animals (CEUA process no. 472–2023).

Twenty-two male and female dogs participated, selected for elective sterilization surgery from the local community, whose owners authorized and signed the Informed Consent Form (ICF). Inclusion criteria were age between 1 and 5 years, weight between 6 and 15 kg, and ASA I classification, according to the American Society of Anesthesiologists. The dogs were considered healthy, as they did not present alterations in the general physical examination and complementary tests (blood count, alanine aminotransferase, creatinine, albumin, electrocardiogram, echocardiogram, and serology for Leishmaniasis—indirect ELISA for detection of anti-Leishmania IgG antibodies).

Exclusion criteria were any alteration in complementary exams, previous cardiovascular alterations, dogs reactive in serology for leishmaniasis, irascibility, estrus, pregnancy, overweight, and obesity. In addition to those dogs whose owner did not sign the ICF.

### 2.2. Acquisition of Baseline Electrocardiographic Parameters

The acquisition of parameters at baseline moment (MB) was performed during a preanesthetic consultation, after anamnesis, general physical examination and collection of blood tests. In order to minimize the effects of stress and physical restraint on the variables of interest, the dogs were tranquilized with butorphanol (0.25 mg/kg/IM) (Butorfin® 1%, Vetnil, Louveira, Brazil) and remained for 15 min in a calm, air-conditioned (23°C) ambulatory.

Subsequently, the dogs were gently positioned in right lateral decubitus, on a non-conductive surface, and the thoracic electrodes were connected to the olecranon region and the pelvic electrodes to the patellar ligament region on both limbs. Immediately afterwards, 70% alcohol was instilled to provide good contact between the electrodes and the skin, allowing the recording of the electrocardiographic tracing (50 mm/s and standard calibration of 10 mm = 1 mV) for 3 min using a 6-lead digital electrocardiograph. (ECGPC TEB, Tecnologia Eletrônica Brasileira, São Paulo, Brasil). Then, the electrocardiographic record was stored for later analysis.

### 2.3. Acquisition of Parameters After Dexmedetomidine Administration

The acquisition of the parameters of interest after the administration of dexmedetomidine moment (MDEX) was performed on the day of elective sterilization of the dogs, within a maximum period of 5 days after the measurement of the basal parameters.

To this end, after trichotomy and antisepsis, venous access was obtained in the cephalic vein with an appropriately sized catheter, which was occluded with an adapter plug and filled with heparinized solution. Then the dogs remained for 15 min in a calm, air-conditioned (23°C) ambulatory to minimize the effects of stress.

They were then gently positioned in the right lateral decubitus position on a nonconductive surface, and an intravenous bolus of dexmedetomidine was administered (Dexdomitor 0.5 mg/mL, Zoetis, São Paulo, Brasil) (2 μg/kg, em dois minutos), immediately afterwards, with the aid of a syringe pump (MedRena SP50 Vet, Shenzhen, China), continuous infusion of dexmedetomidine was started at a rate of 2 μg/kg/hora [23]. After 10 min of continuous infusion, blood pressure was measured and electrocardiographic parameters were collected as previously described.

### 2.4. Electrocardiographic Parameters

All measurements were performed in lead II. (50 mm/s, 10 mm = 1 mV), in triplicate, by the same evaluator, namely, heart rate (HR), duration and amplitude of the P wave, duration of the QRS complex, amplitude of the R wave and T wave, duration of the PR interval and the QT interval. The corrected QT interval (QTc), based on the RR intervals, was calculated using the following equation: QTc = QT − 0.087 ∗ (RR − 1000) [17, 24].

Still in lead II, the durations of the first 20 RR intervals, of sinus origin, were used to calculate the vasovagal tone index (VVTI) according to the following equation: VVTI = NL ∗ [VAR ∗ (R-R1 − R-R20)], where NL = natural logarithm and VAR = variance [25, 26].

After that, the mean electrical axis of the QRS complex and the T wave, in the frontal plane, were determined by the graphical method through the measurement of the net deflection in leads I and III. [27, 28]. With these values, through the absolute difference between the mean electrical axis of the QRS complex and the T wave, the frontal QRST angle was obtained. Whenever the difference exceeded 180°, it was represented as the subtraction of the absolute value from 360° [29].

### 2.5. Blood Pressure Measurement

Different from the acquisition of MB electrocardiographic parameters, the dogs' systolic (SBP), mean (MAP), and diastolic (DBP) blood pressure was measured by the oscillometric method on the day of the experiment. After 15 min of acclimatization, as previously described, the dogs were positioned in the right lateral decubitus position and, using an appropriate cuff, oscillometric blood pressure was measured using a multiparameter monitor. (uMEC12 Vet, Mindray Medical, Gurugram, Índia). For this purpose, 5 blood pressure measurements were performed, and the highest and lowest values were excluded. From this, the final value of SBP, MBP, and DBP was determined by the average of the three remaining values. The same was done in MDEX.

### 2.6. Sedation Score

The sedation score was assessed at the beginning of the MDEX. A sedation scale adapted from Tisotti et al. [18] was used., scored from 0 to 3, where the intensity of the physical restraint necessary to keep the patient in lateral decubitus and the dog's level of awareness in relation to the environment were considered (Appendix A).

### 2.7. Statistical Analysis

From a Q-Q plot comparing the quantiles of the residuals with the quantiles of a theoretical normal distribution, the normality between the residuals of the presented response variables was demonstrated. The linearity of the points suggests that all the study variables are normally distributed.

In compliance with this assumption, the variables were submitted to the paired *T*-test to compare the means between MB and MDEX. A difference was considered significant when *p* value < 0.05. The Spearman test was used to investigate correlations between HR and electrocardiographic parameters (*p* value < 0.05). To interpret the magnitude of Spearman's correlation, the following classification was adopted: correlation coefficients < 0.3 (poor), 0.3 and 0.5 (reasonable), 0.6 and 0.8 (moderately strong), and > 0.8 (very strong).

The data were analyzed using the computer program R v4.3.3 [30].

## 3. Results

All 22 dogs underwent monitoring and measurement of electrocardiographic parameters in the MB and MDEX. Of these, 10 (45.4%) were males and 12 (54.6%) were females, of no defined breed, weighing 9.04 ± 1.54 kg (mean ± SD). The electrocardiographic parameters are shown by the mean followed by the lower and upper quantiles in Table 1.

**Figure 1:**
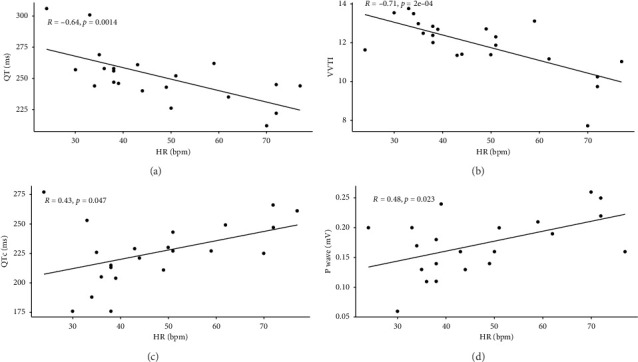
Scatterplots representing significant negative correlation between heart rate, QT interval (a) and vasovagal tone index (b). And significant positive correlation between heart rate, QTc interval (c) and P wave amplitude (d).

All electrocardiographic variables were tested for correlation with HR. Only the amplitude of the P wave, the VVTI, and the QT and QTc intervals showed a significant correlation with HR (Figure 1). The other variables studied did not show a significant correlation with HR.

**Figure 2:**
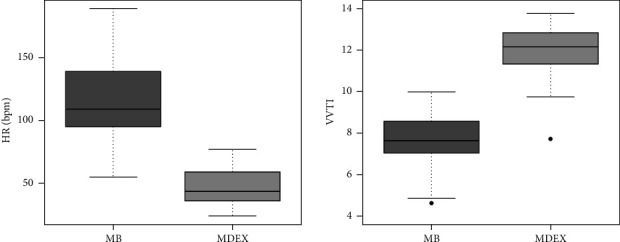
Box plot depicting the medians, interquartile ranges and amplitude of heart rate (HR) and vasovagal tonus index (VVTI) in healthy dogs (*n* = 22) at MB (baseline moment) and MDEX (after bolus of dexmedetomidine 2 µg/kg IV, followed by continuous rate infusion 2 µg/kg/h).

A significant reduction in HR and a significant increase in VVTI were observed between MB and MDEX (Figure 2), with a moderately strong negative correlation between both variables (*R* = 0.71 and *p* < 0.01). Sinus arrhythmia (SA) was the predominant heart rhythm of dogs in MB, while sinus bradycardia (HR < 60 bpm) [28] was predominant in 17 dogs (88.3%) in MDEX. At this point, only 5 dogs (22.7%) had a HR higher than 60 bpm, with a maximum average HR value of 77 bpm.

**Figure 3:**
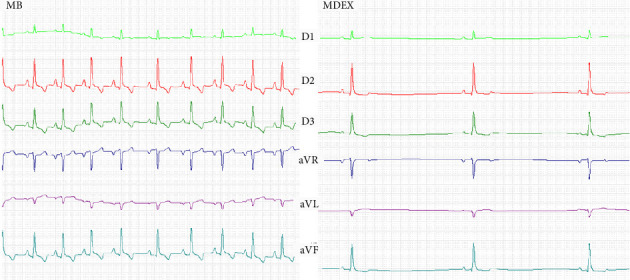
Electrocardiographic tracing of the 6 leads (50 mm/s and 10 mm = 1 mV) of a study dog before (MB) and after 10 min (MDEX) of bolus (2 μg/kg/IV) followed by IC (2 μg/kg/hour) of dexmedetomidine. Note a marked reduction in HR (mean HR in MB = 160 bpm and mean HR in MD = 60 bpm) with the presence of sinus pauses (1400 ms) and inversion of T wave polarity in MDEX.

There was no significant change in relation to the amplitude of the T wave (*p*=0.130), however, 5 dogs (22.7%) presented changes in the polarity of the T wave after administration of dexmedetomidine (1 dog from positive to negative and 4 dogs from negative to positive) (Figure 3).

Regarding the PR interval (ms), 5 dogs (22.7%) presented an increase in MDEX greater than 130 ms, characteristic of first degree atrioventricular block (AVB) [28]. Among these dogs, the minimum PR interval value was 133 ms and the maximum value was 189 ms. Another 2 dogs (9%) presented second degree AVB on MDEX, classified as atypical Mobitz type I 2nd degree AVB. In 12 dogs (54.5%), the presence of sinus pauses longer than 2000 ms was identified during the first 20 beats of sinus origin in the MDEX, where the longest sinus pause was 3443 ms in one of the dogs. No significant differences were observed between the electrocardiographic indicators of ventricular arrhythmogenesis TpTe (evaluated in lead II) and frontal angle QRST.

In turn, there was a significant increase in the QT interval, however, the QTc interval reduced significantly and showed a significant positive reasonable correlation in relation to HR (*R* = 0.43 and *p*=0.047). On the other hand, the QT interval showed a significant and negative moderately strong correlation in relation to HR (*R* = −0.67 and *p*=0.001).

Blood pressure was measured in 12 of the 22 dogs, 6 (50%) males and 6 (50%) females, of no defined breed, weighing 8.8 ± 1.67 (Table 2). And no significant differences were observed between MB and MDEX for SBP, MAP, and DBP.

## 4. Discussion

The study showed that after 10 min of intravenous administration of dexmedetomidine (2 μg/kg followed by IC 2 μg/kg/hour), significant changes occur in the electrophysiological and functional parameters of the myocardium of healthy dogs, with a 59% reduction in HR, in addition to an increase in the conduction time of the electrical impulse in the myocardium.

The electrocardiographic study in MB was performed by calming the dogs with butorphanol, an agonist-antagonist opioid with low hemodynamic impact [28, 31]. Valuing the obtaining of the parameters of interest with minimal influence of stress and enabling the execution of the echocardiographic examination to rule out morphofunctional alterations of the myocardium. Therefore, to prevent any type of drug interaction and provide an isolated evaluation of the hemodynamic effects of dexmedetomidine in dogs, the parameters were collected in the MB within 1–5 days before the MDEX was performed.

Thus, in the MB, the dogs presented a satisfactory degree of tranquilization and allowed the reliable collection and performance of electrocardiographic parameters and echocardiographic examination, which after administration of butorphanol (0.25 mg/kg/IM), remained within the reference range for the size of dogs [28]. The degree of sedation was also satisfactory in MDEX (33.3% score 1% and 66.6% score 2); however, with significant hemodynamic repercussions.

In the present study, the significant increase in the duration of the P wave, PR interval, and QRS complex in MDEX demonstrates that intravenous administration of dexmedetomidine results in a longer duration of atrial and ventricular depolarization time. It is known that the main component related to the duration of the P wave is left atrial activation [32]; therefore, we can admit that the intravenous administration of dexmedetomidine promotes a delay in the intra-atrial conduction time of the electrical impulse. This can be reinforced by the fact that these parameters do not present a significant correlation with HR. Therefore, we can conclude that the significant change in these parameters between the moments is not dependent only on the reduction in HR.

On the other hand, we observed a positive correlation between HR and P wave amplitude, corroborating the fact that there is a significant reduction in this variable in the presence of sinus bradycardia resulting from the administration of dexmedetomidine. Added to this is the significant increase in the duration of the QT interval, which suggests a longer duration of the monophasic action potential, that is, of the depolarization and repolarization phases of the ventricular myocardium [22, 28].

However, even with the presence of an increase in the aforementioned electrocardiographic parameters, only the duration of the P wave and the QT interval exceeded the estimated reference limit for the size of awake dogs [28].

Houve também, incremento no tempo de condução das vias intermodais, demonstrado em nosso estudo pela presença do AVB de 1° grau em 22.5% dos cães no MDEX. In contrast, only 9% of dogs in the MDEX presented moments of total refractoriness of the atrioventricular node, identified by the presence of second-degree AVB, which may suggest that dexmedetomidine has a low direct effect on the atrioventricular node, at the proposed dose [17]. It is important to emphasize that no dog in this study presented any type of atrioventricular conduction block in the MB, with the AVBs found in MDEX being a consequence of the administration of dexmedetomidine.

In addition to the conventional parameters of duration and amplitude of electrocardiographic waves, we chose to perform two parameters that have recently demonstrated potential as markers of ventricular arrhythmias in dogs during the progression of structural heart diseases, TpTe, and fQRSTa [33, 34]. To the authors' knowledge, there are still no reports of the measurement of these parameters in dogs undergoing anesthesia, sedation or tranquilization. Our study did not demonstrate a significant difference in TpTe and fQRSTa after 10 min of intravenous administration of dexmedetomidine, and neither parameter showed a significant correlation with HR. However, new studies should be conducted to determine the real potential of this drug to generate ventricular arrhythmias.

In this context, there was a significant increase in the QT interval in MDEX, which could suggest an arrhythmogenic potential of dexmedetomidine [35]. Lima et al. [22] also observed an increase in this variable after administration of dexmedetomidine (0.5 µg/kg/IM) associated with methadone (0.3 mg/kg/IM) or midazolam (0.3 mg/kg/IM) in dogs. As well, when associated with IC dexmedetomidine (0.5 μg/kg/hour) with propofol, 27% of dogs present QT segment prolongation [15]. However, when we corrected the QT interval value, based on the RR interval duration value, we noticed that the QTc interval reduced significantly in MDEX. Thus, it is possible to associate that the increase in the depolarization and repolarization time of the myocardium of dogs in MDEX may be related to the reduction in HR [36, 37].

This is corroborated by the significant positive correlation between HR and the QTc interval in our study. Meanwhile, the QT interval shows a significant negative correlation with HR. That is, with the bradycardia resulting from the administration of dexmedetomidine there is a tendency for a reduction in the QTc interval and an increase in the QT interval.

Sinus bradycardia was the predominant rhythm in MDEX and can be explained by the increase in parasympathetic tone, also demonstrated by the significant increase in VVTI in MDEX. [17, 25, 26]. This hypervagotonia has a negative chronotropic effect on dogs subjected to intravenous administration of dexmedetomidine, which showed a 59% reduction in HR and an increase in the duration of electrical impulse propagation in the myocardium in MDEX. This can be seen by the strong positive correlation between FC and VVTI. Another indication of increased parasympathetic tone in MDEX is the presence of sinus pauses and the consequent significant increase in the duration of RR intervals [38].

The sinus pauses observed in our study, lasting up to 3.4 ms, fall within a range that may be considered concerning, especially when associated with clinical signs or hemodynamic instability. In veterinary literature, pauses longer than 2 s are often regarded as abnormal and potentially dangerous, as they may lead to reduced cardiac output and risk of syncope [28]. However, the clinical significance of these pauses depends not only on their duration but also on their functional impact on the patient. This should be taken into consideration when using dexmedetomidine under the conditions demonstrated here.

SBP and MAP values showed a significant increase after 18 min of bolus (2 µg/kg/IV) of dexmedetomidine [10], time that was not contemplated in our study. Where SBP, PAM, and DBP do not show significant difference between MB and MDEX. On the other hand, increased PVR was observed after 5 and 15 min of intravenous administration of dexmedetomidine (bolus of 1.2 μg/kg followed by IC of 1.25 μg/kg/hour) in dogs anesthetized with propofol [15]. Therefore, as we did not measure PVR, it is not possible to determine whether there was an increase in this variable without reflecting a significant increase in SBP, MBP and DBP. Therefore, it is possible that the baroreflex mechanism may have contributed to the reduction in HR in our study.

The study was conducted in healthy dogs and its findings should not be interchanged with those of patients with heart disease. However, it is important to highlight the magnitude of the hemodynamic changes described here and the possible consequences that the use of dexmedetomidine, at the proposed dose, may have on dogs with heart disease.

This study has some limitations, such as not using invasive blood pressure monitoring and obtaining MB data days before MDEX data. Another limitation could be the sample size. Without a power analysis or sample size determination study, there's a risk that the sample might be underpowered. This limitation should be acknowledged, and results should be interpreted with caution.

## 5. Conclusion

Intravenous bolus administration of dexmedetomidine (2 μg/kg) in healthy dogs results in increased parasympathetic tone with significant reduction in HR and increased electrical impulse conduction time in the myocardium.

## Figures and Tables

**Table 1:** Electrocardiographic parameters, mean (Qi-Qs), of healthy dogs (*n* = 22) before (MB) and after 10 min (MDEX) of continuous rate infusion of dexmedetomidine (bolus of 2 µg/kg IV followed by IC at a rate of 2 µg/kg/hour).

Parameters	MB		MDEX		*p* value
HR (bpm)	117	(93–139)	48	(36–57)	< 0.001
VVTI	7.61	(7.07–8.55)	11.9	(11.35–12.81)	< 0.001
Average RR intervals (ms)	517	(381–610)	1295	(989–1492)	< 0.001
P wave duration (ms)	44	(42–47)	51	(49–54)	< 0.001
P wave amplitude (mV)	0.21	(0.16–0.23)	0.17	(0.14–0.20)	0.023
QRS duration (ms)	62	(59–69)	67	(63–71)	0.002
R wave amplitude (mV)	1.1	(0.84–1.40)	1.28	(0.99–1.54)	0.008
T wave amplitude (mV)	0.29	(0.15–0.39)	0.35	(0.19–0.46)	0.130
PR interval (ms)	95	(83–103)	126	(114–130)	< 0.001
QT interval (ms)	207	(193–227)	252	(243–258)	< 0.001
Interval QTc (ms)	249	(237–262)	226	(211–246)	0.023
QRS axis (°)	64	(52–77)	65	(66–77)	0.753
TpTe (ms)	29	(27–31)	30	(28–32)	0.200
fQRSTa	79	(17–153)	62	(12–119)	0.183

*Note:* VVTI: vasovagal tone index; TpTe: distance from the peak to the end of the T wave; fQRSTa: frontal QRST angle, MB: basal moment (dogs subjected to tranquilization with butorphanol 0.25 mg/kg).

Abbreviation: HR, heart rate.

**Table 2:** Blood pressure (oscillometric method), mean ± SD, of dogs (*N* = 12) before (MB) and after 10 min (MDEX) of bolus (2 μg/kg/IV) followed by continuous infusion (2 μg/kg/hour) of dexmedetomidine.

Parameters (mmHg)	MB	MDEX	*p* value
SBP	144 ± 18.5	133 ± 19.7	0.12
MAP	107 ± 17.6	110 ± 18.7	0.67
DBP	90 ± 26.0	99 ± 17.6	0.30

*Note:* MB: baseline moment (dogs awake).

Abbreviations: DBP, diastolic blood pressure; MAP, mean arterial pressure; SBP, systolic blood pressure.

## Data Availability

The data that support the findings of this study are available from the corresponding author upon reasonable request.

## Appendix A: Sedation Score (Adapted From [18])

Score 0: Active and alert, with no apparent sedation and/or excitable-dysphoric (excited, anxious, difficult to contain when lying down, very interactive and responsive, vocalizing, reactive to voice and touch);

Score 1: Calm, minimal sedation (quiet, still alert and aware of surroundings, slight resistance to restraint in lateral decubitus, moderate response to voices and touch);

Score 2: Very calm, with moderate sedation (quiet, relaxed, minimal physical restraint required to maintain lateral recumbency, mild response to voices or touch);

Score 3: Deep sedation (silent, very relaxed, no need for restraint for position changes, does not offer resistance to maintaining lateral position, does not respond to voice or touch).
